# DeepNavNet: Automated Landmark Localization for Neuronavigation

**DOI:** 10.3389/fnins.2021.670287

**Published:** 2021-06-17

**Authors:** Christine A. Edwards, Abhinav Goyal, Aaron E. Rusheen, Abbas Z. Kouzani, Kendall H. Lee

**Affiliations:** ^1^School of Engineering, Deakin University, Geelong, VIC, Australia; ^2^Department of Neurologic Surgery, Mayo Clinic, Rochester, MN, United States; ^3^Mayo Clinic Graduate School of Biomedical Sciences, Mayo Clinic, Rochester, MN, United States; ^4^Mayo Clinic College of Medical Scientist Training Program, Mayo Clinic, Rochester, MN, United States; ^5^Department of Physiology and Biomedical Engineering, Mayo Clinic, Rochester, MN, United States

**Keywords:** deep brain stimulation, deep learning, human-machine teaming, landmark localization, neuroimaging, neuronavigation, neurosurgery planning

## Abstract

Functional neurosurgery requires neuroimaging technologies that enable precise navigation to targeted structures. Insufficient image resolution of deep brain structures necessitates alignment to a brain atlas to indirectly locate targets within preoperative magnetic resonance imaging (MRI) scans. Indirect targeting through atlas-image registration is innately imprecise, increases preoperative planning time, and requires manual identification of anterior and posterior commissure (AC and PC) reference landmarks which is subject to human error. As such, we created a deep learning-based pipeline that consistently and automatically locates, with submillimeter accuracy, the AC and PC anatomical landmarks within MRI volumes without the need for an atlas. Our novel deep learning pipeline (DeepNavNet) regresses from MRI scans to heatmap volumes centered on AC and PC anatomical landmarks to extract their three-dimensional coordinates with submillimeter accuracy. We collated and manually labeled the location of AC and PC points in 1128 publicly available MRI volumes used for training, validation, and inference experiments. Instantiations of our DeepNavNet architecture, as well as a baseline model for reference, were evaluated based on the average 3D localization errors for the AC and PC points across 311 MRI volumes. Our DeepNavNet model significantly outperformed a baseline and achieved a mean 3D localization error of 0.79 ± 0.33 mm and 0.78 ± 0.33 mm between the ground truth and the detected AC and PC points, respectively. In conclusion, the DeepNavNet model pipeline provides submillimeter accuracy for localizing AC and PC anatomical landmarks in MRI volumes, enabling improved surgical efficiency and accuracy.

## Introduction

Imaging technologies such as magnetic resonance imaging (MRI) provide visualization of brain structures that enable neurosurgeons to plan accurate and safe surgical trajectories (Edwards et al., 2017). In MRI-guided functional neurosurgery, image space is registered to a stereotactic head frame with a built-in three-dimensional (3D) coordinate system using stereotactic planning software (Edwards et al., 2018). This software allows surgeons to view the neuroimaging data to derive 3D coordinates of the brain target(s) to plan the surgical trajectory path(s). This approach has been predominantly used to implant electrodes for deep brain stimulation and to deliver focused ultrasonic ablation, both of which provide therapeutic relief of debilitating movement and psychiatric disorders (Elias et al., 2016; Edwards et al., 2017, 2018).

Although MRI technologies (1.5 and 3 Tesla) have improved over the last decade, visibility of deep intracranial target nuclei remains poor due to imaging protocols that do not provide requisite image resolution and contrast. As such, it is standard practice to use “indirect” targeting methods to locate DBS targets in preoperative MRI scans. DBS targets are typically indirectly identified by manually locating visible anatomical landmarks, i.e., anterior and posterior commissure points (AC and PC), and using these reference points to align the patient’s preoperative MRI scan to a stereotactic brain atlas. In contrast, “direct” targeting methods may be used when targeted structures are visible and directly identifiable in preoperative images, e.g., acquired using special MRI sequences designed to target specific structures, acquired in an ultra-high field MRI scanner, or in visualizations created through multimodal fusion techniques (Cho et al., 2010; Grewal et al., 2018, 2020; Hartmann et al., 2019). Despite advances in visualizations and direct targeting methods, manual localization of AC and PC points remain standard practice and ACPC alignment is valuable beyond targeting such as for multimodal visualizations for pre- and post-operative assessments (Isaacs et al., 2020). The locations of deep brain surgical targets are estimated by defining a distance from the mid AC-PC point and crosschecked against the registered atlas. Targets include various neurosurgical structures including the subthalamic nucleus (STN) for Parkinson’s disease, ventral intermediate nucleus for essential tremor, globus pallidus internus for dystonia, and the centromedian-parafascicular complex for Tourette’s syndrome. For example, the STN is typically located 5 mm inferior, 1–2 mm posterior, and 9–12 mm lateral from the mid AC-PC point (Slavin et al., 2006). Therefore, accurate identification of the AC and PC points is of vital importance for safe and efficacious outcomes.

A review of factors impacting DBS targeting accuracy indicate that human errors during image-guided planning contribute to an overall average localization error of 1–2 mm (Li et al., 2016). Error in the localization of the AC and PC anatomical landmarks has contributed to misidentification of the target nuclei and misalignment with the stereotactic brain atlas, leading to surgical error (Pallavaram et al., 2008). As a secondary verification, intraoperative electrophysiologic microelectrode recording is performed. This method increases the risk of adverse events such as intracerebral hemorrhage and seizures, especially if initial AC-PC determinations are incorrect (Hariz and Fodstad, 1999; Binder et al., 2005). Thus, a novel system that provides more accurate and reliable AC-PC localization would improve functional neurosurgery. With the revival of artificial intelligence (AI), sparked by deep learning methods demonstrating human-level performance in computer vision applications, it is now possible to conceive an AI-powered DBS surgical targeting system that augments surgical planning by the neurosurgery team (Panesar et al., 2020).

Early methods to automatically localize anatomical landmarks relied on the identification of surrounding anatomical structures, e.g., ventricles, corpus callosum and fornix. These methods used low-level image processing techniques (e.g., edge detection, histogram analysis, and region growing) coupled with anatomical knowledge-based rules to conduct scene analysis (Verard et al., 1997; Bhanu Prakash et al., 2006). Furthermore, they depend on intensity-based image processing techniques (e.g., binarization) to accurately identify regions of interest, and are thus considered antiquated in light of more modern robust methods. These traditional image processing approaches were superseded by atlas registration and machine learning based techniques (D’Haese et al., 2005; Pallavaram et al., 2009). Ardekani and Bachmann introduced a template model to characterize the displacement of the AC and PC points from an additional more prominent anatomical landmark (i.e., midbrain-pons junction), and demonstrated increased accuracy over methods that relied on primitive image processing techniques (Ardekani and Bachman, 2009). A random forest regression approach demonstrated increased accuracy from millimeters to submillimeter localization errors compared to atlas-based methods for detecting AC and PC points within a limited set of 100 T1-weighted MR volumes acquired uniformly from an institution (Liu and Dawant, 2015). These regression forests were built upon texture-based features (i.e., local binary patterns), which have largely been replaced by more robust deeply-learned features in the computer vision community (LeCun et al., 2015).

Since the resurgence of artificial intelligence in the past decade, deep learning based methods continue to rapidly evolve and dominate computer vision applications for natural images and videos. Deep regression approaches are commonly used for facial landmark detection, human pose estimation and image registration applications (Lathuiliere et al., 2019). Although deep learning methods applied in the medical imaging domain were lagging behind, they are gaining momentum (Akkus et al., 2017; Hinton, 2018; Korfiatis and Erickson, 2019). Variations of fully convolutional network (FCN) deep learning architectures (e.g., U-Net) have achieved state-of-the-art results for image segmentation and localization within medical imaging (Falk et al., 2019). Payer et al. augmented an FCN architecture with a spatial configuration network (SCN) module which preserves the geometric relationships between landmarks, and enables more accurate heatmap regression to localize landmarks where large annotated medical image datasets are less readily available (Payer et al., 2016, 2019). Zhang et al. (2017) proposed a cascaded two-stage multi-task deep convolutional neural network architecture to simultaneously localize hundreds of anatomical landmarks in medical images where a limited amount of training data is available (Zhang et al., 2017). In particular, Zhang et al. detected 1200 landmarks in 700 MRI scans, with a mean error of 2.96 mm (Zhang et al., 2017). Given the achievements of using deep learning methods for challenging neuroimaging tasks, it is expected that deep learning approaches would achieve increased accuracy for the presumably less complex task of localizing midline points, e.g., the AC and PC points.

The aforementioned deep learning based methods have demonstrated state-of-the-art and promising results for a variety of medical image modalities and applications. Even so, these methods neither report submillimeter accuracy in localization of landmarks in neuroimaging data, nor report the localization of AC and PC points as required for targeted functional neurosurgery planning. Here, we present the development and validation of a novel deep learning-based heatmap regression pipeline that is built upon an implementation of a 3D residual network (Liu et al., 2018) that demonstrates submillimeter accuracy in localization of the AC and PC landmarks within MRI volumes.

## Materials and Methods

### Data Preparation

Experiments were conducted using two datasets collated from publicly available T1-weighted 1.5-Tesla (T) MRI volumes, which we will refer to as ACPC-MRI-1 and ACPC-MRI-2. Each MRI scan is 256 × 256 × 150 voxels with a cubic millimeter resolution per voxel. The first dataset, ACPC-MRI-1, is comprised of 908 MRI volumes across 687 subjects from the following publicly available data: Open Access Series of Imaging Studies (OASIS) (Marcus et al., 2007) and Mindboggle-101 (Klein et al., 2017). The OASIS dataset is comprised of scans for subjects aged 18 to 96 years, with a subset of subjects aged 60 years and older who were diagnosed with the onset of dementia to moderate Alzheimer’s disease (Marcus et al., 2007). Two to four additional scans are included for the subset of 100 subjects with dementia and 20 healthy subjects (Marcus et al., 2007). The MindBoggle dataset includes T1-weighted MRI volumes from 101 healthy subjects (Klein and Tourville, 2012). The second dataset, ACPC-MRI-2, contains 220 volumes across 158 subjects from the OASIS-3 dataset (LaMontagne et al., 2019). The AC and PC anatomical landmarks were manually localized for a total of 1,128 MRI annotated volumes across ACPC-MRI-1 and ACPC-MRI-2 datasets.

**FIGURE 1:**
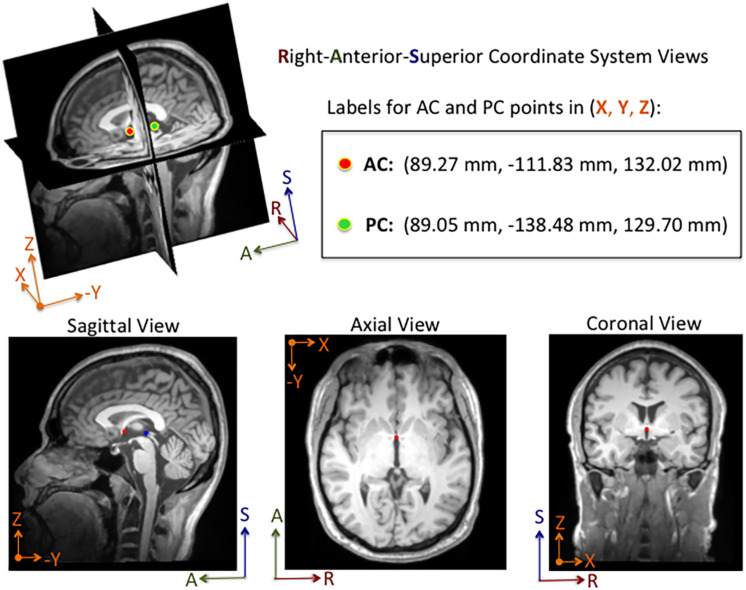
Example of an MRI volume annotated using 3D Slicer software. The volume is displayed in Right-Anterior-Superior (RAS) coordinate space. The annotator manually adjusts fiducial markers overlaid on the AC and PC points from linked 2-D views. For this subject, the AC and PC points are both visible in the same sagittal slice, whereas only the AC point is visible in the axial and coronal slices shown here. Depending on the orientation of the brain within the MRI volume, the AC and PC points are sometimes both visible within a single sagittal and an axial slice. The 3D coordinates for each point are displayed in millimeters in the Cartesian X-Y-Z space. The labels and corresponding 3D coordinates are saved to a fiducial comma-separated values (.fcsv) file.

An annotation protocol was established to label the AC and PC anatomical landmarks for each T1-weighted MRI volume. To streamline the annotation process, each volume was aligned to a common MRI template using a rigid registration approach, which is publicly available in the Advanced Normalization Tools (ANTs) software toolkit (Avants et al., 2011). Each normalized MRI volume was imported into the publicly available 3D Slicer Tool^1^ and viewed with a predefined AC and PC template overlaid on the volume (Kikinis et al., 2014; Fedorov et al., 2016). Using the 3D Slicer Tool, each annotator manually fine-tuned reference point locations to the posterior and anterior edges of the AC and PC points, respectively, and saved the 3D coordinates for each point to a fiducial markup file per MRI volume (see Figure 1). Without fine tuning the locations, the average 3D Euclidean distances between initial locations provided by our template and our ground truth locations of AC and PC points were 6.91 ± 5.50 mm and 6.44 ± 4.89 mm (*N* = 1128), respectively. For the ACPC-Dataset-1, a team of five annotators ranging from novice to an experienced neurosurgeon labeled the AC and PC points for a total of 908 T1-weighted MRI volumes across 687 subjects. Multiple annotators labeled a subset of the MRI scans (*N* = 274), and the resulting 3D coordinates for each landmark were averaged and used as ground truth. For quality control, when multiple labels were available for a given point, the 3D Euclidean distance between points was calculated The average AC and PC distance, across annotators, was 1.14 ± 0.57 mm (2.92 mm max error) and 1.04 ± 0.53 mm (2.94 mm max error), respectively. For distances greater than 2.0 mm, the labels were visually evaluated and adjusted by an expert annotator. For MRI volumes where only a single annotation was available and the annotator was considered a novice, the label was visually inspected and adjusted as needed. Approximately 10% of the labeled points were re-adjusted manually within the Slicer Tool by an expert-level annotator. For ACPC-Dataset-2, an expert-level annotator labeled the AC and PC landmarks of all the volumes, using the same annotation protocol described for ACPC-Dataset-1.

The DeepNavNet models described in this paper regress from an input MRI volume to an output heatmap volume with spheres centered around 3D coordinates of the AC and PC landmarks within each volume. Therefore, training data preparation included an additional step of creating heatmap volumes with Gaussian spheres, with sigma set to 3, centered around 3D coordinates of the AC and PC landmarks within each MRI volume. Each heatmap was created with the same dimensions as their corresponding MRI volume, and the voxel intensity values were normalized to a maximum value of 1. The ACPC-MRI-1 dataset was used for training, validation and testing, whereas the ACPC-MRI-2 data was used for additional testing only. As such, heatmap creation was only necessary for the ACPC-MRI-1 dataset.

### Deep Learning Software Framework and Computational Resources

All experiments were conducted using the open source NiftyNet (0.6.0) framework for medical image analysis built on Google’s TensorFlow (1.15.0) deep learning framework, on a Lambda Labs TensorBook laptop with an NVIDIA GeForce GTX 1070 with 32 GB of memory (Gibson et al., 2018). DeepNavNet’s localization model was built upon NiftyNet’s regression application, the original implementation of which utilize an iterative sampling method to regress from MRI T2-weighted images to CT images (Berger et al., 2017). To create DeepNavNet, this application was adapted to train models to learn a mapping from MRI volumes to volumetric heatmaps concentrated around corresponding AC and PC anatomical landmark locations. NiftyNet configuration files were modified to experiment with different training paradigms to create an optimized model to automatically locate the AC and PC with the greatest accuracy, and a post-processing Python script was created to extract the 3D coordinates for the AC and PC landmarks from heatmap volumes.

### Deep Learning Architecture and Training Experiments

DeepNavNet models were trained using a 3D residual network architecture that was originally created to segment volumetric medical images, with demonstrated success parcellating anatomical structures within neuroimaging data (Li et al., 2017). This 20-layered network includes dilation to widen the receptive field to provide voxel context and more spatial resolution compared to other convolutional neural networks used for segmentation applications, e.g., (U-Net) (Ronneberger et al., 2015; Li et al., 2017). Also, the network includes residual connections, which are known to enable effective training of deep networks by preserving and propagating information through the networks (Lundervold and Lundervold, 2019). All experiments used NiftyNet’s built-in implementation of this architecture, which is referred to as *highres3dnet_large* in the software framework.

NiftyNet’s framework includes a patch-based analysis pipeline with different sampling methods to extract windows from input data, as is often required for neuroimaging deep learning applications to counter challenges of high-dimensional data and GPU memory limitations. DeepNavNet builds upon this feature to employ a weighted sampling method with Gaussian sphere heatmaps as sampling priors for the AC and PC locations. This heatmap was created from the initial AC and PC locations in the template used during the manual annotation process previously described. Also, this template heatmap was used as an error map for regression. NiftyNet’s implementation includes an option to fine tune sampling by updating the error maps, however, this was not necessary for our application. DeepNavNet was trained with a spatial window size set to (72, 72, 1) and a random flipping layer set to augment the training data with three orthogonal 2D views for patch analysis. In addition to flipping sampled windows, training data was randomly augmented by up to 10% increases and decreases in spatial volume scaling. For all experiments, the training batch size was set to 64, which is the number of windows processed per iteration.

**FIGURE 2:**
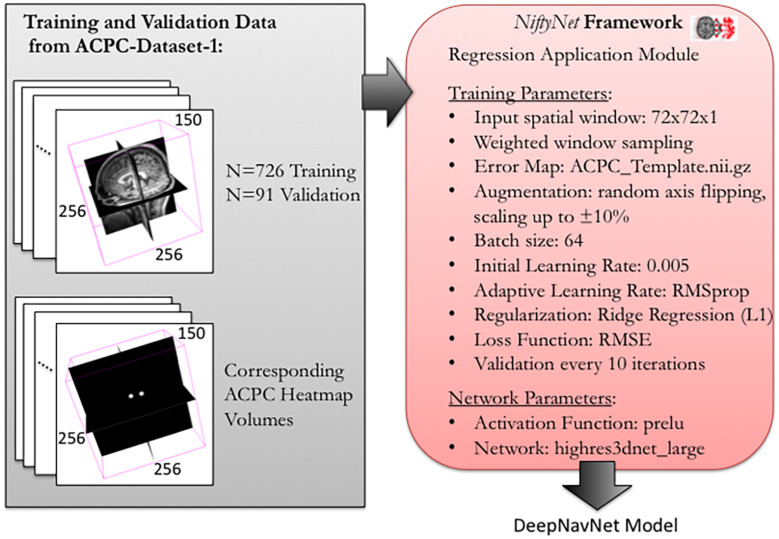
Training and validation of DeepNavNet model using NiftyNet framework. Each input data volume is 256 × 256 × 150 voxels with 1 mm^3^ × 1 mm^3^ × 1 mm^3^ resolution. Key parameters from a customized NiftyNet configuration file are displayed on the right. These parameters were used to create DeepNavNet Model #1 (see Table 1).

To optimize DeepNavNet for automated AC and PC localization, we generated variations using a combination of different training parameters. Loss functions used include the root mean squared error (RMSE) and Huber loss functions (Lathuiliere et al., 2019). Regularization was achieved by using a combination of data augmentation with lasso regression (L1) or ridge regression (L2) regularization methods. Adaptive learning rate techniques used were adaptive moment estimation (Adam) and root mean square propagation (RMSprop), with initial learning rate and weight decay parameters set to 0.005 and 1e-5, respectively (Kingma and Ba, 2015). Each variation of the DeepNavNet model was trained for 2,500 iterations and corresponding learning curves were captured via Tensorboard software. An 80:10:10 ratio was used to separate the ACPC-MRI-1 dataset into subsets of data used for training (*N* = 726), validation (*N* = 91) and testing (*N* = 91). Table 1 provides a list of training experiments that created 8 variations of the DeepNavNet model, which were then tested for accuracy during inference experiments (see Figure 2). Figure 2 also lists the training hyperparameters employed within DeepNavNet, which were optimized during model validation.

**TABLE 1:** DeepNavNet training experiments.

**DeepNavNet Model #**	**Loss function**		**Regularization**		**Adaptive learning rate**	
	**RMSE**	**Huber**	**L1**	**L2**	**Adam**	**RMSProp**
1	✓		✓			✓
2	✓			✓		✓
3		✓	✓			✓
4		✓		✓		✓
5	✓		✓		✓	
6	✓			✓	✓	
7		✓	✓		✓	
8		✓		✓	✓	

### Inference Experiments

Inference experiments were conducted to evaluate model accuracy as measured by calculating the 3D Euclidean distance error between the ground truth and predicted AC and PC coordinates. Initial evaluations of all the models used a test set of 91 volumes from ACPC-MRI-1 dataset, which were volumes not used to train nor validate the models during the training process. If the model was able to detect the AC and PC coordinates with reasonable accuracy, then additional testing was performed with the full-set of 220 volumes from ACPC-MRI-2 dataset, and the resulting 3D Euclidean localization errors were combined to determine the overall accuracy across both datasets (*N* = 311). First, a baseline evaluation was established using the publicly available Automatic Registration Toolkit (ART) software package, which includes an Ardekani and Bachmann model-based approach to detect AC and PC points and a model trained on T1-weighted MRI volumes (Ardekani and Bachman, 2009) (Klein et al., 2009). The acpcdetect module within ART was used to detect the AC and PC locations within the combined dataset of 311 MRI test volumes. A Python script was created to extract the AC and PC 3D coordinates from the output text file per MRI volume.

**FIGURE 3:**
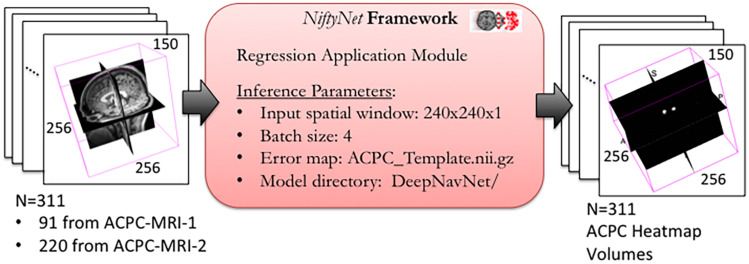
DeepNavNet heatmap regression using NiftyNet Framework for test data.

**FIGURE 4:**
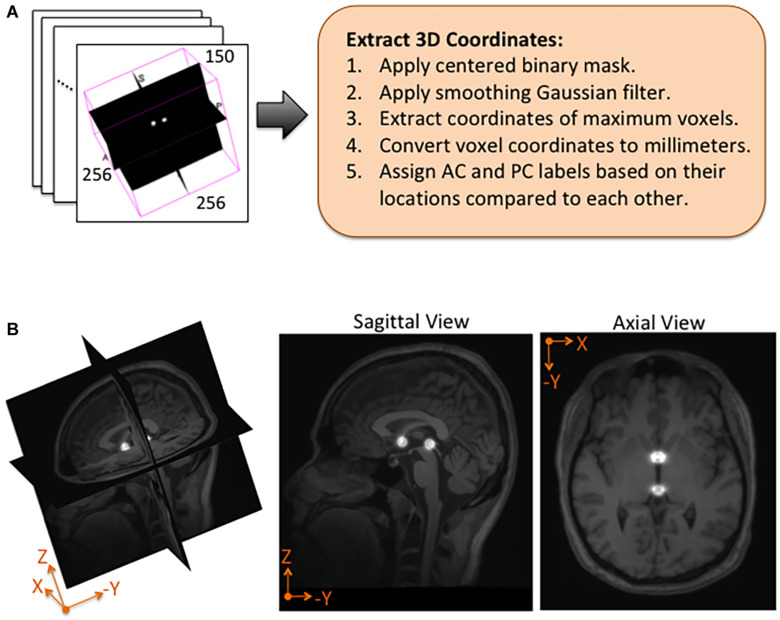
Post-processing and overlay visualizations of a predicted AC-PC heatmap volume. **(A)** The 3D coordinates of AC and PC points are extracted from each predicted heatmap volume. **(B)** Visualizations of a post-processed predicted heatmap overlaid on its corresponding MRI scan. The radius of the spheres is approximately 7.5 mm.

Using NiftyNet software, inference experiments were conducted to test the accuracy of each instantiation of the DeepNavNet model listed in Table 1. The DeepNavNet model predicts a heatmap for each input MRI volume (see Figure 3). A post-processing Python script was created to extract the predicted 3D coordinates for the AC and PC landmarks from the predicted heatmaps. Since input MRI volumes are aligned to a template volume, the AC and PC landmarks are expected to be relatively centered within a region of the volume. Furthermore, there are anatomical constraints regarding the position of the AC and PC with respect to each other. As such, post-processing included applying a binary mask to filter out false positives outside this region of interest, applying a Gaussian filter to smooth the spheres, and assigning AC and PC labels to maximum voxels identified based upon their spatial relationship to each other (see Figure 4).

### Statistical Analysis

Outliers were identified and removed from each model’s localization errors prior to comparison between models to ensure fair comparison. Outliers were defined as those errors which were greater than 3 scaled median absolute deviations (MAD) away from the median of the errors for that model. Scaled *MAD* = c*median(|*A*_*i*_−*median*(*A*)|) for all i in the model, where $c=\frac{-1}{\sqrt{2}*e\,r\,f\,c^{-1}\,\left(1.5\right)}$. After outliers were removed, AC errors across models were compared with a one-way analysis of variance (ANOVA), and post-hoc comparisons between DeepNavNet models and baseline were conducted with one-tailed unpaired t-tests, while post-hoc comparisons between DeepNavNet models were done with two-tailed unpaired t-tests. A Bonferroni correction with 6! = 720 comparisons was applied to each significance level (α = 0.05) to correct for multiple comparisons. P-values less than 0.001 are reported as such rather than reporting the exact value. The same procedure was performed for PC localization errors across all models.

## Results

**FIGURE 5:**
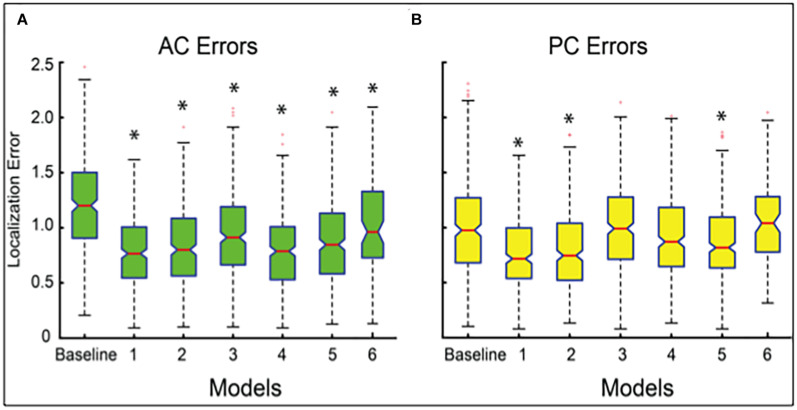
Boxplots of localization errors achieved, measured in millimeters, by the baseline and DeepNavNet models 1-6. Statistical analysis of the baseline and Models 1-5 were conducted using all of the test data (*N* = 311), whereas Model 6 was evaluated using a subset (*N* = 91). Box whiskers span 1.5 times the interquartile range (IQR) in both directions. The 95% confidence interval spans 1.57 times the IQR /√n around the median notch for each box. Non-overlapping notches indicate significant differences between models, and asterisks indicate *p*-values < <0.001 relative to baseline error. **(A)** Boxplots for AC localization errors. **(B)** Boxplots for PC localization errors.

Our best performing DeepNavNet (Model-1) achieved a mean localization error of 0.79 ± 0.33 mm (1.62 mm max error) and 0.78 ± 0.33 (1.66 mm max error) between the ground truth and the detected AC and PC points across *N* = 311 volumes, respectively (see Table 2). A one-way ANOVA was conducted across all models separately for AC and PC localization errors to assess differences in model ability to accurately localize these landmarks. We found that models did perform differently for both AC and PC localization (AC: F = 47, *p* < < 0.001; PC: F = 19, *p* < <0.001). Each DeepNavNet instantiation was then compared directly to the baseline model, separately for AC and PC localization error. Models 1-6 were found to significantly outperform the baseline model for accurately localizing the AC landmark (one-tailed unpaired t-tests, all adjusted *p* < <0.001; see Figure 5A). For localizing the PC landmark, models 1, 2, and 5 were found to significantly outperform the baseline model (one-tailed unpaired t-tests, all adjusted *p* < <0.001; see Figure 5B).

**TABLE 2:** Average 3D AC and PC localization errors of baseline and DeepNavNet models, measured in millimeters.

**Model # (Description, N = # test MRIs)**	**AC Mean ± Std Dev (Max)**	**PC Mean ± Std Dev (Max)**
0 (Baseline)	1.22 ± 0.44 (2.57)	1.00 ± 0.45 (2.31)
1 (RMSE-L1-RMSprop) (*N* = 311)	**0.79 ± 0.33 (1.62)**	**0.78 ± 0.33 (1.66)**
2 (RMSE-L2-RMSprop) (*N* = 311)	0.84 ± 0.35 (1.91)	0.80 ± 0.36 (1.84)
3 (Huber-L1-RMSprop) (*N* = 311)	0.93 ± 0.40 (2.08)	1.00 ± 0.38 (2.14)
4 (Huber-L2-RMSprop) (*N* = 311)	0.81 ± 0.35 (1.85)	0.92 ± 0.39 (2.01)
5 (RMSE-L1-adam) (*N* = 311)	0.87 ± 0.38 (2.05)	0.86 ± 0.35 (1.87)
6 (RMSE-L2-adam) (*N* = 91)	1.01 ± 0.43 (2.08)	1.03 ± 0.42 (2.07)
7 (Huber-L1-adam) (*N* = 91)	X	X
8 (Huber-L2-adam) (*N* = 91)	X	X

*An “X” denotes that the Model did not predict heatmaps with AC and PC landmarks highlighted. Models 0–5 were evaluated using the full test set (*N* = 311), whereas Models 6–8 were only evaluated on the first test set (*N* = 91) due to their reduced performance. The bolded values correspond to the best performing model as measured by the lowest mean localization errors.*

To assess model precision, we assessed the spread of AC and PC localization error distributions across our models with outliers kept in the data. These outliers represent failures of the model to reliably locate the AC and PC landmarks across trials. The number of outliers and the standard deviation of the models with outliers kept are presented in Table 3. The source of large outliers for the baseline method is unknown and inconsistent; however, visual inspection indicates that it is unable to reliably detect AC and PC points within images with advanced signs of dementia, e.g., atrophy, enlarged ventricles, and asymmetries across the hemispheres. In contrast, the DeepNavNet models produced a fewer number of outlier errors and the magnitude of the errors were much smaller. Visual inspection of the MRI scans does not indicate the cause of the outlier errors, however, the results were consistent across the DeepNavNet models.

**TABLE 3:** Number of outliers and the resulting standard deviation and maximum error measured in millimeters with the outliers included for the baseline and our DeepNavNet models.

**Model # (Description, N = # test MRIs)**	**AC # Outliers, Std Dev (Max)**	**PC # Outliers, Std Dev (Max)**
0 (Baseline)	11, 5.01 (62.59)	10, 3.45 (49.76)
1 (RMSE-L1-RMSprop) (*N* = 311)	**3, 0.38 (3.25)**	**6, 0.36 (2.00)**
2 (RMSE-L2-RMSprop) (*N* = 311)	2, 0.38 (2.94)	1, 0.36 (1.91)
3 (Huber-L1-RMSprop) (*N* = 311)	4, 0.42 (2.38)	0, 0.38 (2.14)
4 (Huber-L2-RMSprop) (*N* = 311)	4, 0.39 (2.31)	3, 0.41 (2.74)
5 (RMSE-L1-adam) (*N* = 311)	1, 0.39 (2.49)	4, 0.37 (2.30)
6 (RMSE-L2-adam) (*N* = 91)	6, 11.39 (79.40)	4, 8.09 (72.65)

*The bolded values correspond to the best performing model as measured by the lowest mean localization errors.*

**FIGURE 6:**
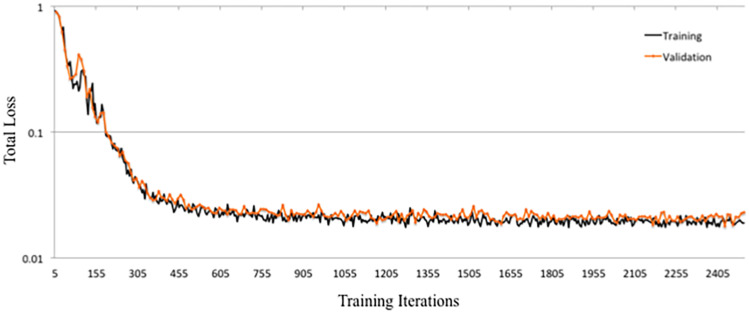
Training and validation learning curves for DeepNavNet Model-1.

DeepNavNet Model-1 was trained using the RMSprop adaptive learning rate method, coupled with RMSE loss function and L1 regression regularization methods, and demonstrated submillimeter accuracy that is significantly better than the baseline method. In addition to further evaluating models against a second independent dataset (i.e., ACPC-MRI-2), the learning curves for training and validation data were examined to assess how well the learned model generalizes to previously unseen data. The pattern of progression of the training and validation learning curves for our overall best performing model DeepNavNet-1 indicates that the learned model does not overfit the training data and is expected to maintain reasonable accuracy across datasets (Lundervold and Lundervold, 2019; see Figure 6).

**FIGURE 7:**
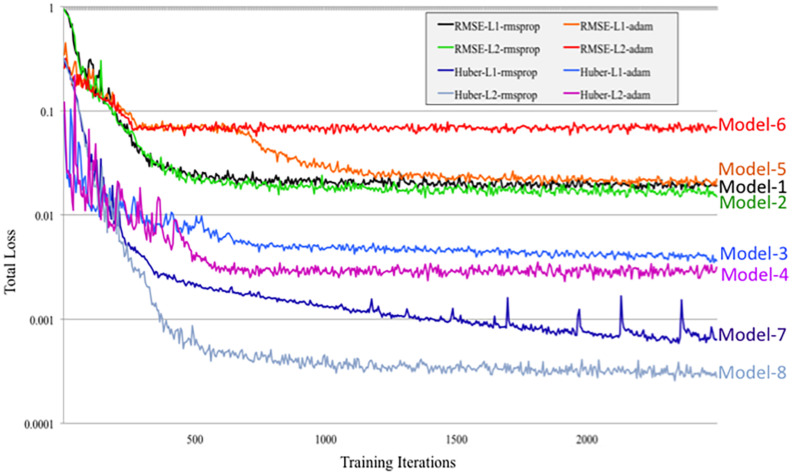
Tensorboard training learning curves for DeepNavNet Models 1-8 using hyperparameters described in Table 1.

Another instantiation of our model (DeepNavNet Model-2) achieved comparable accuracy to DeepNavNet Model-1 for PC localization; however, even though it also achieved submillimeter accuracy, its AC localization error was greater than Model-1. DeepNavNet Model-1 and Model-2 were trained using the same hyper parameter settings, however, the latter used L2 regression regularization instead of L1 regression. The training curves for the DeepNavNet Model-1 and Model-2 follow a very similar stable and gradual progression toward their minimum total loss values across training iterations, with the total loss values for Model-2 being slightly lower than Model-1 (see Figure 7).

Similarly, DeepNavNet Model-3 and Model-4 were trained using the RMSprop adaptive learning rate technique with L1 and L2 regression regularization techniques, respectively. Although they achieved a significant boost in accuracy compared to baseline (Model-0), they were less accurate than DeepNavNet Model-1 and Model-2. Using the Huber loss function, in contrast to the RMSE function, produced the less accurate DeepNavNet Model-3 and Model-4. Further, their PC localization errors were greater than their AC localizations errors.

DeepNavNet Model-5 significantly outperformed the baseline model and achieved submillimeter accuracy for localizing AC and PC, however, it underperformed compared to DeepNavNet Model-1 and Model-2. The training curve for DeepNavNet Model-5 shows that the model required more training iterations to reach minimum loss values, after which the model then tracks similarly to DeepNavNet, Models 1 and 2 (see Figure 7).

DeepNavNet Models 6 to 8 were not evaluated on the ACPC-MRI-2 test set due to the poor quality of their predicted heatmaps from the ACPC-MRI-1 test set. DeepNavNet Model-6 outperformed the baseline for AC localization, however, it was less accurate compared to the other DeepNavNet models. Further, assessment of its total loss across training iterations shows that Model 6 abruptly reached its minimum total loss values sooner, with total loss values larger than high performing instantiations of DeepNavNet models (see Figure 7). This is an indication that the initial learning rate (i.e., 0.005) in our configuration may need adjustment. DeepNavNet Models 7 and 8 failed to predict heatmap volumes with the AC and PC points highlighted as indicated by “X” values in Table 2. All of the DeepNavNet models that used the Huber loss function (Models 3, 4, 7 and 8), rather than the RMSE loss function, exhibited a lower total loss across training iterations, however, that is not an indication that the model is able to effectively learn to regress to heatmaps with AC and PC points highlighted (see Figure 7).

In short, our final resulting DeepNavNet pipeline used our best performing DeepNavNet model (Model-1) that was trained using an RMSE loss function with L1 regression regularization and RMSprop adaptive learning rate techniques. Our resulting DeepNavNet pipeline significantly improved the accuracy of automatically detecting the 3D coordinates for AC and PC points, compared to the baseline method. Intermediate versions of our pipeline that used other versions of our DeepNavNet model had mixed results and overall underperformed compared to our final DeepNavNet model (Model-1).

## Discussion

We described the development and evaluation of our novel deep learning-based pipeline that simultaneously detects AC and PC anatomical landmarks with submillimeter accuracy. A core component of this pipeline is the DeepNavNet model which was successfully trained to predict a heatmap with the AC and PC landmarks identified. A key advantage of this method is that one model has been able to detect both AC and PC landmarks within the same predicted heatmap, while preserving the spatial relationship between the landmarks. Furthermore, since all image volumes were normalized to the same template MRI volume with known AC and PC locations, their AC and PC landmarks are expected to be identified within a known region of interest. The DeepNavNet pipeline demonstrated that it is able to automatically identify and fine-tune predicted AC and PC locations with submillimeter accuracy. Post-processing to extract the 3D coordinates for each landmark within the heatmap volumes was relatively simple since the regions of interest were confined to regions around the initial AC and PC template locations. More sophisticated methods to extract centroid coordinates of other anatomical structures from volumetric heatmaps could be considered in future applications, such as a DeepNavNet pipeline to automatically locate specific deep brain stimulation targets.

A key component of our research included the creation of a large-scale annotated dataset of MRI volumes, which were critical for training, validating and testing our models, and will be invaluable for follow-up research within the neuroimaging community. We manually annotated the AC and PC landmark locations for 1,128 1.5-T MRI volumes collated from publicly available data collected from multiple institutions which were acquired from healthy subjects to those with varying degrees of cognitive decline. The anatomical brain structures of subjects with advanced stages of dementia were visibly different from relatively healthy subjects, e.g., enlarged ventricles, atrophy, and asymmetries. As such, manual identification of the AC and PC points took longer, while also providing a more diverse dataset for training and evaluating more robust models. Even so, it is expected that the DeepNavNet models and pipeline would require fine-tuning for different types of MRI scans and other neuroimaging modalities.

The focus of this work was to develop a deep learning based pipeline that demonstrates consistent submillimeter accuracy for detecting AC and PC points, across a large-scale diverse dataset of 1.5-T MRI volumes, as a first step toward an automation pipeline to enable a modernized human-machine teaming approach to surgery planning. Further improvements to accuracy may be achieved by experimenting with other loss functions, such as an adaptive loss function designed for landmark localization (Teixeira et al., 2019). Although we achieved superior accuracy results, the practical implementation of this work will require further research to reduce the overall computation time. In particular, the heatmap inference time per MRI scan is a few minutes, whereas the post-processing step to extract the coordinates is approximately a second per MRI scan. We experimented with multiple combinations of different training parameters, however, further research could leverage techniques that learn optimal hyperparameters (Liaw et al., 2018). Also, meta-learning approaches to identify optimal parameters may reduce the size of the resulting models and enable a more efficient network for inference (Vanschoren, 2018). Furthermore, with the rise of edge computing, there is growing interest in improving the efficiency of deep neural networks to enable deployment of such technologies on edge devices. Methods to prune deep neural networks are expected to enable more efficient models (Blalock et al., 2020). In addition, in April 2020, the creators of NiftyNet framework announced that they are shifting toward an open-source PyTorch-based deep learning framework for medical imaging called Medical Open Network for AI (MONAI).^2^ Future experiments to advance DeepNavNet would likely benefit from moving to the MONAI framework as well since it is expected to gain momentum in the deep learning medical imaging community. Another machine learning approach that is gaining momentum across multiple application domains, including landmark detection in medical images, combines deep learning and reinforcement learning methods – i.e., deep reinforcement learning (DRL) (Mnih et al., 2015; Francois-Lavet et al., 2018; Sutton and Barto, 2018). In contrast to supervised deep learning approaches, which learn to minimize a loss function based on labeled training data, a DRL artificial agent learns to maximize an award function by trial-and-error as it navigates the data environment in a sequential Markov decision process (MDP) framework. Ghesu et al. (2016) introduced an approach that used a DRL agent to locate anatomical landmarks in medical image volumes. Building upon this research, DRL methods continue to advance and show promise as an effective strategy for real-time navigation to landmarks within medical images (Ghesu et al., 2017, 2019; Al and Yun, 2019; Alansary et al., 2019). Even so, establishing an effective decision space and award function that reliably converges is non-trivial for DRL approaches in dynamic environments, and is especially a barrier for using such agents where consistently accurate and safe results are required (Dulac-Arnold et al., 2019). A multi-agent collaborative DRL approach, introduced in 2019, achieved an average localization error of 0.93 ± 0.18 and 1.05 ± 0.25 mm for AC and PC points, respectively (Vlontzos et al., 2019). Although our current supervised deep learning approach is more accurate, there is an acceleration of innovation in DRL methods and such artificial agents are expected to improve through experience as they navigate through more neuroimaging data, such as our ACPC-MRI -1 and ACPC-MRI-2 datasets.

Our method described here focused on identifying AC and PC points as a necessary first step toward aligning to a stereotactic atlas. However, the regression pipeline that we have developed and validated can be applied toward segmentation of a wide range of neural and non-neural structures. The heatmap is a relatively straightforward approach to generate probabilistic distributions of target region locations, enabling rapid model development and training. Post-processing algorithms for region coordinate extraction can then employ more advanced segmentation methods, such as spherical Hough transforms or an additional convolutional neural network. Our model presented herein provides a framework upon which future parcellation networks can be developed. Additional exploration will be needed to optimize our method for other biological structures (e.g., hyperparameter tuning, model architecture, etc.).

Another approach to consider beyond improved indirect targeting using AC and PC landmarks is to employ deep learning methods to directly register a subject’s MRI data to a stereotactic atlas, followed by an indirect targeting process (Sutton and Barto, 2018). In addition, ultra high-field 7.0 T MRI scanners are becoming clinically available at a limited number of large medical institutions (e.g., Mayo Clinic, Rochester, MN, United States), and provide unprecedented high-resolution imaging of deep brain structures (Cao et al., 2018). As such, this technology is expected to enable direct targeting for deep brain stimulation and other therapies. Furthermore, they will provide invaluable data to improve and enable AI-powered surgical planning systems that would learn to safely navigate to deep brain stimulation targets for optimal clinical results. We anticipate that our model will benefit from the incorporation of ultra-high field imaging via direct localization of target nuclei and pathology.

## Conclusion

Our novel DeepNavNet pipeline automatically identifies the 3D coordinates of AC and PC anatomical landmarks in MRI volumetric data with state-of-the-art submillimeter accuracy, as required for image-guided targeted neurosurgery planning procedures. This technology is a first step toward automating surgical planning to improve surgical accuracy and efficiency.

## Data Availability Statement

Publicly available datasets were analyzed in this study. This data can be found here: https://www.oasis-brains.org and https://mindboggle.info/data.html.

## Ethics Statement

Ethical review and approval was not required for the study on human participants in accordance with the local legislation and institutional requirements. Written informed consent for participation was not required for this study in accordance with the national legislation and the institutional requirements.

## Author Contributions

CE designed the methodology and executed the experiments, performed data collation and annotation, developed the DeepNavNet software pipeline, conducted analyses of experimental results, and drafted and revised the manuscript. AG annotated data, conducted statistical analyses, and edited the manuscript. AR annotated data and edited the manuscript. AK provided guidance and edited the manuscript. KL annotated data, provided guidance, and edited the manuscript. All authors contributed to the article and approved the submitted version.

## Conflict of Interest

The authors declare that the research was conducted in the absence of any commercial or financial relationships that could be construed as a potential conflict of interest.

## Footnotes

1 3D Slicer Tool available from http://www.slicer.org (accessed May 2018).

2 Medical Open Network for Artificial Intelligence available from https://monai.io
